# Supplementary material for: Theoretical and Experimental Assay of Shock Experienced by Yeast Cells during Laser Bioprinting
Source: Int J Mol Sci. 2022 Aug 29;23(17):9823. doi: 10.3390/ijms23179823 (PMC9456252; doi:10.3390/ijms23179823)
Supplement: Supplementary file 1 [file ijms-23-09823-s001.zip › ijms-1863683-supplementary.pdf]

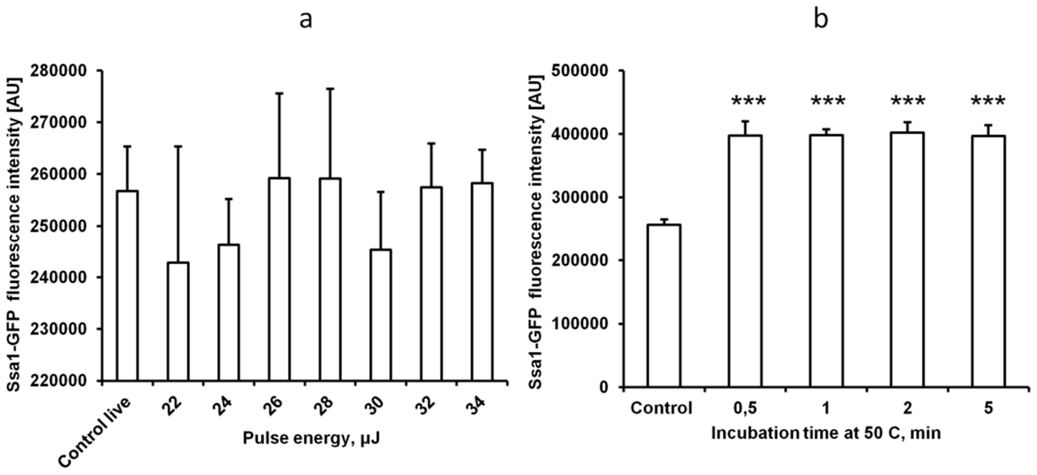

Figure SI. Cells that were grown to lower densities did exhibit an increase of Ssa1-GFP levels in response to 50 C heat shock , but did not do so in response to LIFT . (a) Relation between level of median GFP fluorescence and the laser pulse intensity (in PI-negative cells with noticeable GFP fluorescence) ($p \geq 0{,}1$, Student's t-test) (b) Fluorescence of yeast cells producing Ssa1-GFP after treatment with the indicated temperature ($p \leq 0{,}01$ (***), Student's t-test)
